# Acute Toxicity, Immunotoxicity and Allergenicity of Protease Complex Obtained from Micromycete *Sarocladium strictum*

**DOI:** 10.3390/pharmaceutics13101660

**Published:** 2021-10-11

**Authors:** Svetlana Alipkina, Elena Kornienko, Denis Nalobin, Alexander Osmolovskiy

**Affiliations:** Biological Faculty, M.V. Lomonosov Moscow State University, 119234 Moscow, Russia; aljnka-93@mail.ru (E.K.); denis.nalobin@gmail.com (D.N.)

**Keywords:** immunotoxicity, allergenicity, acute toxicity, protease complex, micromycete, *Sarocladium strictum*, thrombolytics, plasminogen activators

## Abstract

The different effects on animals of the thrombolytic protease complex of the new producer *S. strictum* 203 were studied. The tests of acute toxicity, immunotoxicity and allergenicity should conclude that the studied proteolytic complex is safe for medical usage. For the intravenous and the intraperitoneal routes of administration, the maximum tolerated dose and the median lethal dose were not determined.

## 1. Introduction

One of the main goals of modern medicine is the diagnosis and treatment of conditions caused by thrombosis. Thrombolytic agents, which can be used in therapy, are proteolytic enzymes with an activity similar to one of haemostatic system proteases [1]. Such activity was found in proteases present in cultures of micro-organisms. The preparations obtained on the basis of cultures of micro-organisms can be used as therapeutic agents for the treatment conditions caused by thrombosis [2]. Extracellular proteolytic enzymes of micromycetes can act on haemostatic system proteins as activators or direct fibrinolytic agents [3,4]. The proteases of *Sarocladium strictum* 203 (VKM F-4845D) showed a direct fibrinolytic activity and a plasminogen-activating effect (indirect fibrinolytic activity) [5]. In this regard, the study of the properties of proteases from this producer is essential.

The study of acute toxicity, immunotoxicity and allergenicity is very important to conduct before non-clinical studies. This allows us to draw general conclusions about the safety of the studied substance and to save further time and money. It is important at the early stages of the development of a drug to assess its prospects and the possibility of its further use for the treatment of humans.

This work aims to study allergenicity, immunotoxicity, and acute toxicity of proteinases of the micromycete *S. strictum* 203.

## 2. Materials and Methods

### 2.1. S. strictum Extracellular Proteinase Preparation

Micromycete *S. strictum* 203 were supplied by the Department of Microbiology, Moscow State University and deposited in the All-Russian collection of microorganisms (number VKM F-4845D). The strain was maintained in tubes with slant agar. To receive the substance *S. strictum* 203 was cultured in submerged conditions. A culture of the micromycete was grown on Czapek’s slant medium for 7 days, after that, spores were washed by inoculum media and transferred to the medium for 48–72 h. Part of the biomass (10% *v*/*v*) was transferred to the fermentation medium for 96–120 h at a temperature of 28 °C on a rocker at 220 rpm. The medium for enzyme biosynthesis had the following composition (in g/L): sucrose, 40.0; K_2_HPO_4_, 4.4; NaNO_3_, 19.0; KNO_3_, 2.5; pH 6.5. At the end of the cultivation, the culture liquid was separated from the mycelium by centrifugation (15,000× *g*, 15 min, 4 °C). Proteins from the culture fluid were precipitated by acetone (2 *v*/*v*) cooled to −20 °C, kept for 1 h at 4 °C, and then filtered. The filtered precipitate was dried in a vacuum desiccator over calcium chloride for 3 days. The received substance was a homogeneous yellowish–white powder without smell, well soluble in water. For the received substance, in vitro the tests were carried out as described earlier [6].

### 2.2. Animals

The acute toxicity study was carried out on Wistar rats (10 weeks old, the weight of males 190–220 g, the weight of females 180–200 g) and C57Bl/6 mice (8 weeks old, weight 20–22 g) of both sexes.The study was conducted according to the guidelines of the Declaration of Helsinki, and approved by the Bioethics Committee of M.V. Lomonosov State University (protocol 16-1 dated 14.05.2021).

The allergenicity study was carried out on Wistar rats (8–10 weeks old, the weight of males 190–220 g, the weight of females 180–200 g) of both sexes; CBA mice (6–8 weeks old, weight 20–22 g) of both sexes; CD1 mice (6–8 weeks old, weight 20–22 g) of both sexes; Soviet chinchilla Rabbits (2–2.2 kg) of both sexes.

The immunotoxicity study was carried out on (C57Bl/6xDBA)F1 mice (6–8 weeks old, weight 20–22 g) males.

All rodents were born in the vivarium of Moscow State University and used for studies in vivo. Animals were kept by groups (5 animals per group for mice and 2 animals per group for rats and 1 rabbit per group) under a 12 h light-dark cycle (lights on at 08:00), 30–70% relative humidity and at 20–24 °C. Adult rabbits of either sex weighing 1–1.5 kg were purchased from local market and kept in the vivarium of Moscow State University for one week for acclimatization. Standard food for rabbits and filtered tap water were provided ad libitum.

Animal content and all studies involving animals were carried out in accordance with the rules [7,8]. Substance was dissolved in normal saline (0.9% sterile NaCl solution) and was prepared fresh before administration.

### 2.3. Allergenicity

Allergenicity of substance was studied in four separate allergenicity tests: (1) active cutaneous anaphylaxis in mice; (2) active systemic anaphylaxis test in rats; (3) concanavalin A–induced inflammatory reaction in mice; (4) conjunctive test in rabbits. For carrying out these experiments, Wistar rats of both sexes, CBA mice of both sexes, CD1 mice of both sexes, Soviet chinchilla rabbits of both sexes were used.

### 2.4. Active Cutaneous Anaphylaxis in Mice

The division of animals into groups was performed. The first group included 10 CD1 mice (5 males and 5 females) receiving injections of normal saline. The second group included 10 CD1 mice (5 males and 5 females) receiving injections of substance, which was administered at a dose of 24 mg/kg. The third group included 10 CD1 mice (5 males and 5 females) receiving injections of substance, which was administered at a dose of 240 mg/kg.

First injection was performed subcutaneously, second and third injections were performed intramuscularly into the quadriceps muscle of the thigh (0.2 mL/kg).

An active cutaneous anaphylaxis test was performed on the 14th day after the last substance injection [9,10].

On the last 14th day, the mice have injected with substance intracutaneously (20 μL, 1 mg/kg and 10 mg/kg); the same volume of normal saline was injected intracutaneously to another area of the skin.

To assess the reaction, 20 min after the intracutaneous injection, animals were injected by Evans’ blue solution (1%, 0.2 mL/kg) intravenously. The mean diameter and intensity of the ‘blued’ lesion surrounding the injection sites were assessed 30 min later.

### 2.5. Active Systemic Anaphylaxis Test in Rats

Rats were divided equally into three groups of 10 animals (5 males and 5 females) per group and treated as follows: rats of the first group were injected with normal saline subcutaneously (sc) for three days, the second and the third groups were injected with ovalbumin solution (0.012 mg/kg sc) for three days.

On the 12th day after the last ovalbumin injection rats of the first group were injected with normal saline intraperitoneal (ip) (1 mL/kg) for four days, the second group was injected with substance solution (12 mg/kg ip, 1 mL/kg) for four days, the third group was injected with substance solution (120 mg/kg ip, 1 mL/kg) for four days [11,12].

On the 15th day, an hour after the substance injection, all animals were injected with ovalbumin solution (0.036 mg/kg ip). Thirty minutes after ovalbumin injection, the animals were euthanized (CO_2_ inhalation), and the weights of their small intestine were determined. Then, the small intestine was dried, and the weights were determined. The degree of anaphylaxis development was assessed by the severity of the oedema of the small intestine (by changing the content of the total water in it (by drying to constant mass)).

### 2.6. Concanavalin A-Induced Inflammatory Reaction in Mice

CBA mice were divided into three groups. The first group included 10 mice (5 males and 5 females) receiving injection of normal saline subcutaneously (sc). The second group included 10 mice (5 males and 5 females) receiving injection of substance, which was administered at a dose of 24 mg/kg sc (0.2 mL). The third group included 10 CD1 mice (5 males and 5 females) receiving injection of substance, which was administered at a dose of 240 mg/kg sc (0.2 mL).

One hour after substance injection, all animals received Concanavalin A solution (20 μL, 5 mg/mL) in the pad of the left hind paw. The right hind paw received the same volume of normal saline.

After 1 h of the Concanavalin A injection, the animals were euthanized (CO_2_ in-halation). The weights of both paws were determined. The reaction index was calculated by the following formula:

$$R_{i}=\frac{W_{exp}-W_{cont}}{W_{exp}}\;\times \;100\%,$$

where *Ri*—reaction index, *W_exp_*—the weight of the experimental paw, *W_cont_*—the weight of the control paw.

### 2.7. Conjunctive Test in Rabbits

Soviet chinchilla rabbits (2–2.2 kg) of both sexes were divided equally into two groups of 6 animals (3 males and 3 females) per group and treated as follows: rabbits of the first group were injected with normal saline subcutaneously (sc) for five days, the second group animals were treated with substance (0.5 mL, 6.5 mg/kg sc) for five days.

Fourteen days after the last substance administration, all animals were treated as follows: 1 drop of the substance (100 mg/mL) was dipped in the left eye (in a conjunctival sac), 1 drop of the isotonic sodium chloride solution was dipped in the right eye (in a conjunctival sac).

The state of sclera, cornea, and eyelids was verified. The reaction was determined after 15 min (fast response), 24 and 48 h in accordance with the scale of estimating the ophthalmological indicators [13].

### 2.8. Immunotoxicity

Three following experiments were performed: (1) a delayed-type hypersensitivity test; (2) T-Cell-Dependent Test; (3) phagocytic activity of peritoneal macrophages. For all three tests 90 male (C57Bl/6xDBA)F1 mice were used (30 mice per test).

Mice were divided into three groups. The first group included 30 (15 males and 15 females) mice receiving injection of normal saline subcutaneously (sc). The second group included 30 mice (15 males and 15 females) receiving injection of substance, which was administered at a dose of 20 mg/kg sc (0.2 mL). The third group included 30 mice (15 males and 15 females) receiving injection of substance, which was administered at a dose of 200 mg/kg sc (0.2 mL).

The substance was injected subcutaneously daily for five days (0.2 mL/kg), after which the following tests were performed.

### 2.9. Evaluation of Cellular Immunity in a Delayed-Type Hypersensitivity Test

To perform a Delayed-Type Hypersensitivity Test 30 mice (10 animals (5 males and 5 females) from each group) were injected with a solution of sheep red blood cells (SRBC) (5 × 107 SRBC per animal, 0.2 mL) subcutaneously at the base of the tail. After 5 days the animals were injected with SRBC (5 × 107 SRBC per animal, 50 μL) in the pad of the left hind paw. The right hind paw received the same volume of normal saline.

Finally, 24 h after the injection, the animals were euthanized (CO_2_ inhalation), and the weights of their paws were determined. The reaction index was calculated in the same way as was shown above.

### 2.10. Evaluation of Humoral Immunity in T-Cell-Dependent Test

To perform a T-Cell-Dependent Test 30 mice (10 animals (5 males and 5 females) from each group) were injected with a SRBC intraperitoneally (5 × 107 SRBC per animal, 0.5 mL).

After 5 days of the immunization, the mice were euthanized (CO_2_ inhalation), the spleen was evaluated and weighed, the state of the immune system was evaluated, determining the number of the antibody-producing cells (APC) in the spleen by the plaque-forming cell assay, calculating the modulation index (immune response) compared to the control group [14].

### 2.11. Phagocytic Activity of Peritoneal Macrophages

To perform this test 30 mice (10 animals (5 males and 5 females) from each group) were injected intraperitoneally with 1 mL of ink particles suspension. Ten minutes later the mice were euthanized (CO_2_ inhalation), the peritoneal exudate was isolated. The total number of peritoneal macrophages in the exudate (1 μL) and the number phagocytic cells were counted using a Gorjaev’s chamber.

### 2.12. Acute Toxicity

Acute toxicity was estimated for intraperitoneal (ip) and intravenous (iv) administration to C57Bl/6 mice of both sexes and to Wistar rats of both sexes.

For the iv administration, 40 mice and 40 rats were used.

Mice were divided into 4 groups of 10 animals each. The first group received injection (into the lateral tail vein at a volume of 1 mL/kg) of substance, which was administered at a dose of 0.551 mg/kg. The second group received injection (into the lateral tail vein at a volume of 1 mL/kg) of substance, which was administered at a dose of 1.102 mg/kg. The third group received injection (into the lateral tail vein at a volume of 1 mL/kg) of substance, which was administered at a dose of 2.204 mg/kg. The fourth group received injection (into the lateral tail vein at a volume of 1 mL/kg) of substance, which was administered at a dose of 4.408 mg/kg.

Rats were divided into 4 groups of 10 animals each.

The first group received injection (into the lateral tail vein at a volume of 1 mL/kg) of substance, which was administered at a dose of 0.2755 mg/kg. The second group received injection (into the lateral tail vein at a volume of 1 mL/kg) of substance, which was administered at a dose of 0.551 mg/kg. The third group received injection (into the lateral tail vein at a volume of 1 mL/kg) of substance, which was administered at a dose of 1.102 mg/kg. The fourth group received injection (into the lateral tail vein at a volume of 1 mL/kg) of substance, which was administered at a dose of 2.204 mg/kg.

For the ip administration, 40 mice were used. Animals were divided into 4 groups of 10 animals each. The first group received injection (intraperitoneal at a volume of 1 mL/kg) of substance, which was administered at a dose of 0.551 mg/kg. The second group received injection (intraperitoneal at a volume of 1 mL/kg) of substance, which was administered at a dose of 1.102 mg/kg. The third group received injection (intraperitoneal at a volume of 1 mL/kg) of substance, which was administered at a dose of 2.204 mg/kg. The fourth group received injection (intraperitoneal at a volume of 1 mL/kg) of substance, which was administered at a dose of 4.408 mg/kg.

After iv and ip injections of substance, the animals were observed for 14 days. After this period, all animals were euthanized, and a diagnostic autopsy was performed.

After the administration of the substance, the animals were continuously observed for an hour (visible deviations from normal behaviour, signs of carrying pain and discomfort were recorded), then 5 min every hour for eight hours, signs of the manifestation of intoxication were recorded.

In the next 13 days, animals were examined daily twice a day.

During the study, the following parameters were registered: animal death—daily during the entire observation period; visual inspection of animals (daily); inspection with elements of neurological testing (at least 1 time per week); the weight of animals (at least 1 time per week); consumption of food and water (at least 1 time per week); macroscopic examination of internal organs and histological examination of organs with external pathologies.

The parameters of the general state of animals were recorded visually at least once a week.

The following parameters were studied: activity in the cell, the presence/absence of tremor, the appearance of animals (the state of wool, skin, mucous membranes available for observing), the parameters of the individual emotional status (reaction to moving into a new environment, touch, the presence of urine, defecation during inspection), emotional tension in the cell, simple reflexes (shuddering, turning, corneal, ear). The weight of animals was registered at least once a week. Consumption of water and food was registered at least once a week.

During the autopsy, we investigated: muscular system, internal surfaces, a skull cavity and a brain surface, neck with organs and tissues, chest, abdominal and pelvic cavities with organs and tissues in them. All deviations from the norm were fully described and documented.

### 2.13. Data Analysis and Statistics

For performing the statistical analysis Statistica 7.1 (TIBCO Software Inc., Palo Alto, CA, USA) was used. Kruskal–Wallis ANOVA on ranks test was used for data analysis. Results are expressed as mean of data ± SEM. In all tests, *p* < 0.05 was taken as significant.

## 3. Results

### 3.1. Allergenicity

In the test of active cutaneous anaphylaxis in mice, the mean diameter and intensity of the ‘blued’ lesion surrounding the injection sites were assessed (Table 1). As a result of this test, the statistically significant differences between the control and experimental groups were not identified. Within each group, there were no differences between the stain diameters after the introduction of normal saline and substance. The studied substance does not affect the development of skin anaphylaxis in the presented test and does not show allergenic properties.

In the test of active systemic anaphylaxis in rats, the degree of the development of anaphylaxis was assessed by the severity of the oedema of the small intestine to change the content of the total water in it (method of drying to constant mass) (Table 2).

As a result of this test, the statistically significant differences between the control (the intestinal mass decreased by an average of 41.2% during drying) and experimental groups (mass decreased by an average of 37.5 and 39.1% for groups 12 mg/kg and 120 mg/kg, respectively) are not identified.

Thus, the studied substance does not affect the development of active systemic anaphylaxis in the presented test and does not show allergenic properties.

In the test of Concanavalin A-induced inflammatory reaction in mice, the weights of both paws were determined, and the reaction index was calculated (Table 3).

As a result of this test, the statistically significant differences between the control and experimental groups were not identified. The average reaction index had similar values for all three groups.

Thus, the studied substance does not affect the intensity of the inflammation reaction and does not show allergenic properties.

The Draize Test was used for evaluating the irritant action of the substance in the conjunctive test in rabbits. Three-block parameters were considered to estimate the reaction:

- Cornea (density and length of turbidity of the cornea, the size of the zone involved in the reaction of the cornea); iris (the degree of inflammation of the iris); conjunctiva (hyperaemia of the conjunctiva of the eyelid, the degree of oedema of the conjunctiva, the abundance of separation). Each of the experimental parameters was assigned a score from 0 to 5, depending on the severity of the trait. At both time points, all rabbits, males and females alike, have the indicators of the state of the cornea, the iris and conjunctiva at the original physiological level.

Thus, the analysis of the results of the conjunctival sample showed that the studied substance does not have an irritating effect.

### 3.2. Immunotoxicity

In the humoral immunity in T-Cell-Dependent test in mice, the number of the antibody-producing cells (APC) in the spleen was determined by the plaque-forming cell assay, and the modulation index (immune response) compared to the control group was calculated.

The results of this test were expressed as a number of APC per 1 million nucleus-containing cells in the spleen. It has been established that the administration of the substance does not lead to a change in the mass of the cells of the spleen compared with the control animals (Table 4).

The administration of the substance did not lead to stimulation of humoral immune response in doses of 20 and 200 mg/kg.

As a result of this test, the amount of APC did not increase in a statistically significant manner, and differences between the control and experimental groups were not identified (Table 5). Thus, the analysis of the results of the test showed that the studied substance does not affect the studied indicators of humoral immunity in mice.

In the Delayed-Type Hypersensitivity Test in mice, the reaction index was calculated (Table 6). The analysis of the results of the test showed that the studied substance does not affect the hypersensitivity reaction to SRBC.

In the Phagocytic Activity of Peritoneal Macrophages test the number of peritoneal macrophages and the number of the ink particle-containing (phagocytic) cells were counted. The phagocytic index was calculated (Table 7).

The test results showed that the studied substance does not significantly change the phagocytic activity of peritoneal macrophages at all doses (Table 7). Thus, in all three tests no significant changes in the studied parameters of the immune system in animals receiving the substance were observed.

### 3.3. Acute Toxicity

During the entire period of the experiment (14 days), not a single death was recorded in all groups of animals. No clinical symptoms were observed in the control group. In contrast, increased breathing rate and decreased activity were observed in all animals in the experimental groups immediately after administration. In the first minutes after the intravenous administration of the substance, the rats and mice experienced pain in the area of administration for 30–50 min. Thus, we failed to detect the lethal dose of the studied substance. No lethality was observed at intravenous injection at all doses.

The intraperitoneal administration introduction of the substance also caused a pain syndrome: the animals were assembled into groups in the corner of the cell, they did not move for 10–15 min, then several animals showed classic indicators of strong abdominal pain for 60–90 min.

The general condition of experimental animals was the same as in the control animals during the term of observation (14 days).

Animals showed normal activity in the cell, and there was no tremor, the appearance of animals (the condition of wool, skin, mucous membranes) was normal.

Forage and water consumption were within normal limits.

The faeces and urine were normal consistency, smell, and colour. The reaction of animals to sound and tactile stimuli was adequate. There were no signs of emotional tension.

The weight of animals decreased by the next day after the drug administration by 5–22%, depending on the group, but returned to the initial values a week after the administration. The decrease in body weight was a consequence of stress after the introduction of the substance.

During the macroscopic examination, no visible changes in the internal organs were observed.

Thus, the studied substance in prophylactic and therapeutic doses and in a dose three times greater than the therapeutic did not show toxic effects.

The analysis of the results of the acute toxicity test show that the median lethal dose for the studied substance for the iv and the im administration is more than 4408 mg/kg.

## 4. Conclusions

Summing up the results of all three used tests, we conclude that the studied substance is safe from the point of view of allergenic properties, immunotoxicity and acute toxicity in the conducted experiments on animals. For the intravenous and the intraperitoneal routes of administration, the maximum tolerated dose and the median lethal dose were not determined. That means the proteolytic complex of *S. strictum* 203 may be safe for medical usage and very perspective in the biomedical area.

## Figures and Tables

**Table 1:** The mean diameter of the ‘blued’ lesion surrounding the injection sites in all groups of animals.

Dose, mg/kg	Number of Animals in the Group	Diameter of the ‘Blued’ Lesion, mm	
		Normal saline	Substance
0	10	0.428 ± 0.057	0.472 ± 0.055
24	10	0.745 ± 0.161	0.801 ± 0.171
240	10	0.728 ± 0.154	0.769 ± 0.126

**Table 2:** The content of water in the small intestine in rats immunized with ovalbumin.

Dose, mg/kg	Number of Animals in the Group	The Change of the Content of Water, %
0	10	40.9 ± 1.025
12	10	39.3 ± 1.127
120	10	41.1 ± 0.51

**Table 3:** The influence of substance on the inflammation reaction to Concanavalin A.

Dose, mg/kg	Number of Animals in the Group	Index of the Reaction
0	10	13.7 ± 1.51
24	10	13.55 ± 1.73
240	10	13.81 ± 1.77

**Table 4:** Indicators of body weight, spleen, the number of nucleus-containing cells in immunized mice.

Treatment			Mass of a Spleen, g	Nucleus-Containing Cells mln/Spleen	Number of Animals
Substance		Antigen			
Normal saline		SRBC	0.15 ± 0.012	168.2 ± 13.74	10
Dose, mg/kg	20	SRBC	0.13 ± 0.015	198.5 ± 19.4	10
	200	SRBC	0.16 ± 0.017	177.2 ± 21.2	10

**Table 5:** Effect of the substance on the accumulation of APC in mice.

Treatment			APC, mln/Spleen	The Modulation Index	Number of Animals in the Group
Substance		Antigen	Absolute Value	Absolute Value	
Normal saline		SRBC	4982.5 ± 245.5	29.85 ± 2.17	10
Dose, mg/kg	20	SRBC	4839.2 ± 298.8	24.67 ± 2.45	10
	200	SRBC	4937.5 ± 247.6	27.81 ± 2.86	10

**Table 6:** The effect of the substance on the reaction of the delayed-type hypersensitivity in mice.

Treatment			Weight of the Experimental Paw, mg	Weight of the Control Paw, mg	Index of the Reaction	Number of Animals in the Group
Substance		Antigen				
Normal saline		SRBC	257.2 ± 6.9	177.4 ± 2.3	44.90 ± 4.24	10
Dose, mg/kg	20	SRBC	259.3 ± 6.1	181.3 ± 2.1	43.22 ± 4.27	10
	200	SRBC	260.1 ± 7.2	179.1 ± 2.5	45.63 ± 5.12	10

**Table 7:** Influence of the substance on the phagocytic activity of peritoneal macrophages.

Dose, mg/kg	Number of Animals in the Group	Total Number of Cells in Exudate	Number of Phagocytic Cells
0	10	685 ± 51.6	235 ± 25.9
20	10	688.8 ± 51.2	205.5 ± 24.2
200	10	670 ± 53.8	215 ± 23.6
